# Death With Dignity: A Policy Analysis

**DOI:** 10.1093/geroni/igab046.240

**Published:** 2021-12-17

**Authors:** Zainab Suntai

**Affiliations:** University of Alabama, Tuscaloosa, Alabama, United States

## Abstract

Death with dignity is one of many titles attributed to the practice of providing a patient with terminal illness a means to die in light of extreme suffering as a result of a terminal illness. The purpose of the Oregon Death with Dignity Act is to provide individuals suffering from a terminal illness with the right to make a written request for life-ending medication to end their life in a “humane and dignified manner.” Deborah Stone’s policy goals of equity, efficiency, welfare, security, and liberty provides a framework to analyze whether a policy is doing what it says it wants to do. As such, the goal of this presentation is to apply the policy goals framework to determine if the Death with Dignity Act is accomplishing its stated goals. Applying a theory of the policy process, the Death with Dignity Act was analyzed using the most recently available data from the Oregon Health Authority. Results showed that since the Death with Dignity Act passed in 1997, 2,518 people have received prescriptions for life-ending medications, and of those, 1657 or 66% have used the medication to end their lives. Based on the data aggregated between 1997 and 2019, the Death with Dignity Act has mostly met its purpose of providing individuals with a terminal illness with the right to die on their own terms and with dignity. However, there are still several issues regarding equity, especially for low-income BIPOC populations. Implications for practice, policy and research are discussed.

